# Supplementary material for: A Global Screen for Assembly State Changes of the Mitotic Proteome by SEC-SWATH-MS
Source: Cell Syst. 2020 Feb 26;10(2):133–155.e6. doi: 10.1016/j.cels.2020.01.001 (PMC7042714; doi:10.1016/j.cels.2020.01.001)

Q8IWT6 | LRC8A_HUMAN | LRRC8A KIAA1437 LRRC8 SWELL1 UNQ221/PRO247
Monomer MW [kDa]: 94.199 Monomer expected elution fraction: 41

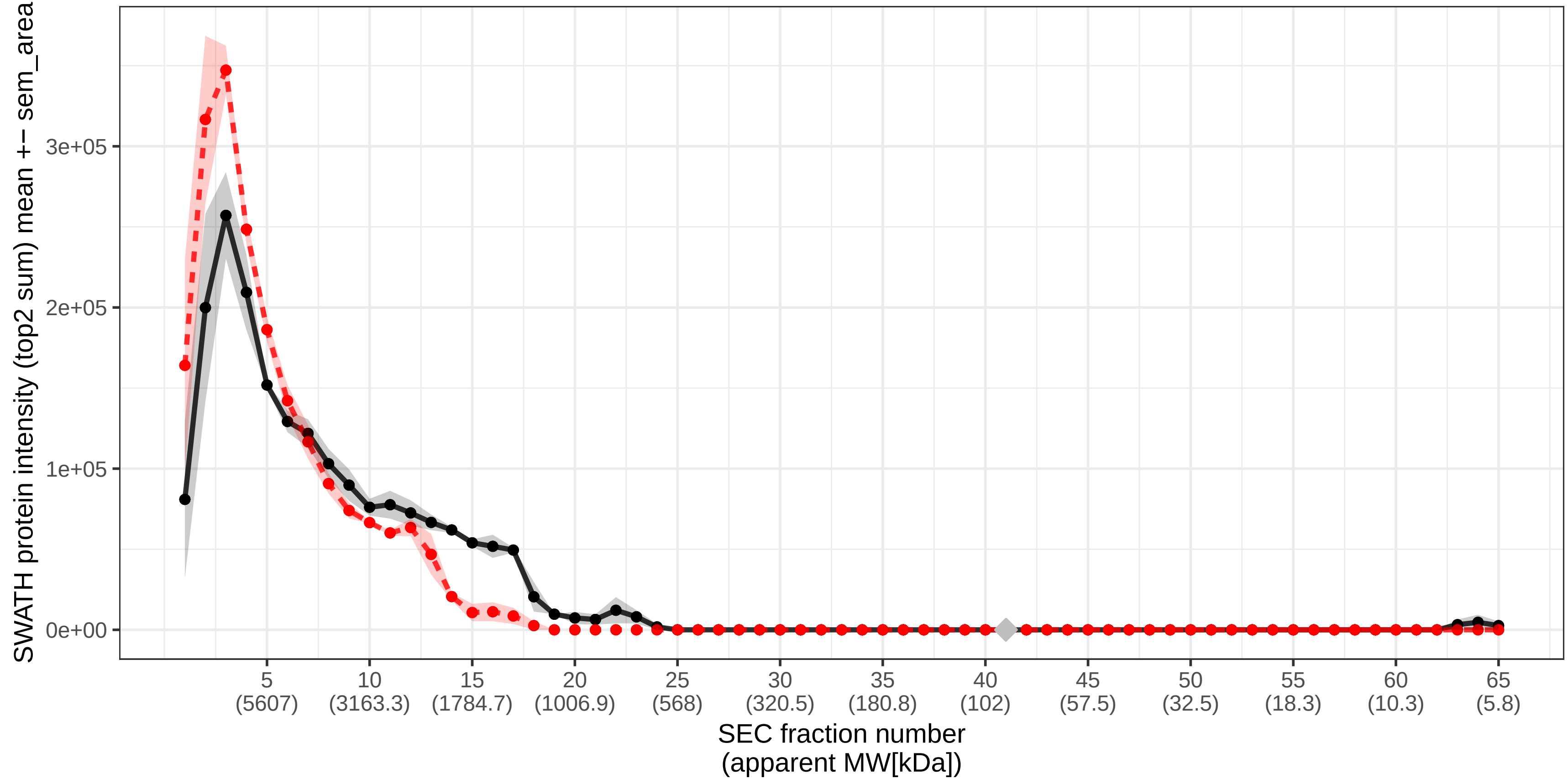

Supplement: Data S1. SEC-SWATH-MS Protein Chromatograms, Related to Figure 1 [file mmc6.zip › SECchrom_Q8IWT6_LRC8A_HUMAN_LRRC8A_KIAA1437_LRRC8_SWELL1_UN.pdf]
